# Anhydrous penta­guanidinium dihydrogen nona­vanado(IV)platinate(IV)

**DOI:** 10.1107/S1600536811049166

**Published:** 2011-11-23

**Authors:** Hea-Chung Joo, Ki-Min Park, Uk Lee

**Affiliations:** a Department of Chemistry, Dongeui University, San 24 Kaya-dong Busanjin-gu, Busan 614-714, Republic of Korea; b Center for Nanobio Chemical Materials (WCU), Department of Chemistry & Research Institute of Natural Science, Gyeongsang National University, Jinju 660-701, Republic of Korea; c Department of Chemistry, Pukyong National University, 599-1 Daeyeon 3-dong, Nam-gu, Busan 608-737, Republic of Korea

## Abstract

The title compound, (CH_6_N_3_)_5_[H_2_PtV_9_O_28_], containing the nona­vanadoplatinate(IV) polyanion, was obtained by hydro­thermal reaction. The polyanion has approximate *C*_2*v*_ symmetry. The two Pt-bound μ_2_-O atoms are protonated in the polyanion. The heteropolyanions form inversion-generated dimers, {[H_2_PtV_9_O_28_]_2_}^10−^, held together by each of the two μ_2_-O—H⋯μ_2_-O and μ_2_-O—H⋯μ_3_-O hydrogen bonds. The guanidinium cations are hydrogen bonded with the μ_2_- and terminal O atoms of the polyanion, connecting the polyanions into a three-dimensional network.

## Related literature

For a structural study of a deca­vanadate, see: Lee (2006 ▶). For the structure of the sodium salt of the title compound, see: Lee *et al.* (2008 ▶). For a related heteropolyoxidometalate, TBA_4_[HTeV_9_O_28_]·2CH_3_CN (TBA = tetra-*n*-butylammonium), see: Konaka *et al.* (2011 ▶). 
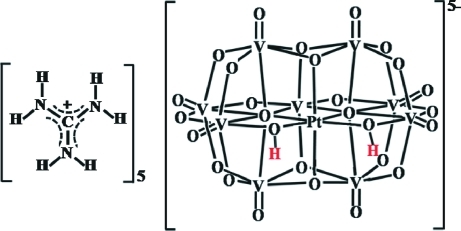


## Experimental

### 

#### Crystal data

(CH_6_N_3_)_5_[H_2_PtV_9_O_28_]
*M* *_r_* = 1404.01
Monoclinic, 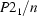

*a* = 12.8861 (3) Å
*b* = 18.5137 (5) Å
*c* = 15.2299 (4) Å
β = 91.143 (1)°
*V* = 3632.67 (16) Å^3^
*Z* = 4
Mo *K*α radiation
μ = 6.15 mm^−1^
*T* = 147 K
0.09 × 0.06 × 0.05 mm

#### Data collection

Bruker SMART APEXII CCD diffractometer
Absorption correction: multi-scan (*SADABS*; Bruker, 2009 ▶) *T*_min_ = 0.187, *T*_max_ = 0.305
35027 measured reflections
9026 independent reflections
7369 reflections with *I* > 2σ(*I*)
*R*_int_ = 0.029

#### Refinement

*R*[*F* ^2^ > 2σ(*F* ^2^)] = 0.029
*wR*(*F* ^2^) = 0.073
*S* = 1.04
9026 reflections
531 parameters
H atoms treated by a mixture of independent and constrained refinement
Δρ_max_ = 2.02 e Å^−3^
Δρ_min_ = −0.75 e Å^−3^

### 

Data collection: *APEX2* (Bruker, 2009 ▶); cell refinement: *SAINT* (Bruker, 2009 ▶); data reduction: *SAINT*; program(s) used to solve structure: *SHELXS97* (Sheldrick, 2008 ▶); program(s) used to refine structure: *SHELXL97* (Sheldrick, 2008 ▶); molecular graphics: *ORTEP-3* (Farrugia, 1997 ▶) and *DIAMOND* (Brandenburg, 1998 ▶); software used to prepare material for publication: *SHELXL97*.

## Supplementary Material

Crystal structure: contains datablock(s) global, I. DOI: 10.1107/S1600536811049166/zl2427sup1.cif

Structure factors: contains datablock(s) I. DOI: 10.1107/S1600536811049166/zl2427Isup2.hkl

Additional supplementary materials:  crystallographic information; 3D view; checkCIF report

## Figures and Tables

**Table 1:** Hydrogen-bond geometry (Å, °)

*D*—H⋯*A*	*D*—H	H⋯*A*	*D*⋯*A*	*D*—H⋯*A*
O7—H7⋯O19^i^	0.73 (6)	2.06 (6)	2.718 (4)	152 (7)
O8—H8⋯O4^i^	0.77 (7)	1.87 (8)	2.626 (4)	165 (8)
N1—H1*A*⋯O26^ii^	0.88	2.11	2.916 (5)	153
N1—H1*B*⋯O17^iii^	0.88	2.18	2.970 (5)	149
N2—H2*A*⋯O25^ii^	0.88	1.99	2.863 (5)	173
N2—H2*B*⋯O12	0.88	2.39	3.105 (5)	138
N3—H3*A*⋯O22^iii^	0.88	2.19	2.973 (5)	148
N3—H3*B*⋯O21	0.88	2.23	3.018 (5)	149
N4—H4*A*⋯O15^iii^	0.88	2.44	3.224 (5)	149
N4—H4*B*⋯O28^iv^	0.88	2.30	2.985 (5)	134
N5—H5*A*⋯O14	0.88	2.06	2.932 (5)	173
N5—H5*B*⋯O28^iv^	0.88	2.10	2.830 (5)	140
N6—H6*A*⋯O12	0.88	2.07	2.899 (5)	156
N6—H6*B*⋯O9^iii^	0.88	1.86	2.737 (5)	171
N7—H7*A*⋯O21^ii^	0.88	2.35	3.084 (5)	142
N7—H7*B*⋯O26^v^	0.88	2.36	3.179 (5)	154
N8—H8*A*⋯O20	0.88	2.12	2.942 (5)	154
N8—H8*B*⋯O13^ii^	0.88	2.04	2.890 (4)	161
N9—H9*A*⋯O11	0.88	2.20	3.025 (5)	157
N9—H9*B*⋯O15^v^	0.88	2.19	2.936 (5)	142
N10—H10*A*⋯O3	0.88	2.07	2.892 (5)	156
N10—H10*B*⋯N7^iv^	0.88	2.62	3.349 (6)	141
N11—H11*A*⋯O23^vi^	0.88	2.40	3.171 (5)	147
N11—H11*B*⋯O23^vii^	0.88	2.06	2.923 (6)	168
N12—H12*A*⋯O26	0.88	2.46	3.159 (5)	137
N12—H12*B*⋯O18^vii^	0.88	2.24	3.063 (5)	157
N13—H13*A*⋯O6	0.88	2.42	3.216 (5)	150
N13—H13*B*⋯O16^viii^	0.88	2.14	2.892 (5)	143
N14—H14*A*⋯O10	0.88	2.02	2.876 (5)	165
N14—H14*B*⋯O14^v^	0.88	2.17	2.947 (5)	147
N15—H15*A*⋯O25^v^	0.88	2.17	3.034 (5)	169
N15—H15*B*⋯O22^viii^	0.88	2.05	2.911 (5)	167

Symmetry codes: (i) 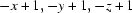; (ii) 
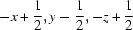
; (iii) 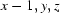; (iv) 
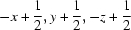
; (v) 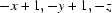; (vi) 
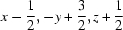
; (vii) 
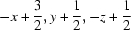
; (viii) 
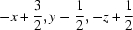
.

**Fig. 1.**
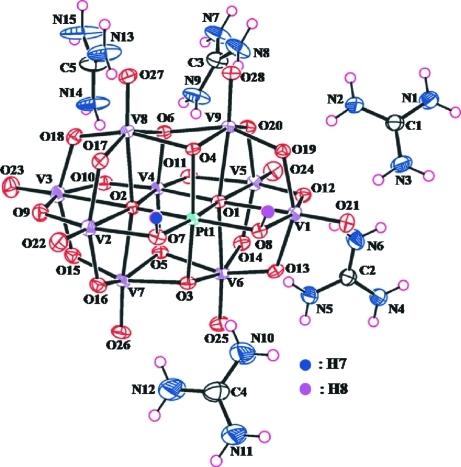
The molecular structure of the title compound with the atom numbering scheme. Displacement ellipsoids are drawn at the 50% probability level. H atoms are presented as small spheres of arbitrary radius.

**Fig. 2.**
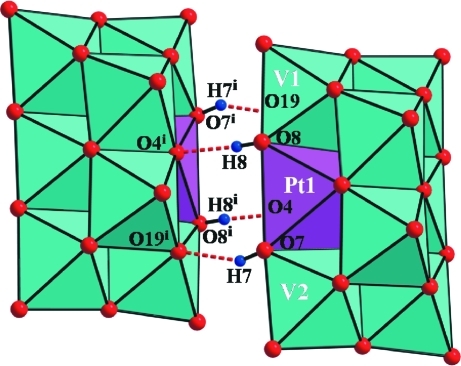
Polyhedral view of the inter-anion hydrogen bonds (dotted lines) in the crystal structure of the title compound. [Symmetry code: (i) -x+1, -y+1, -z+1.]

**Fig. 3.**
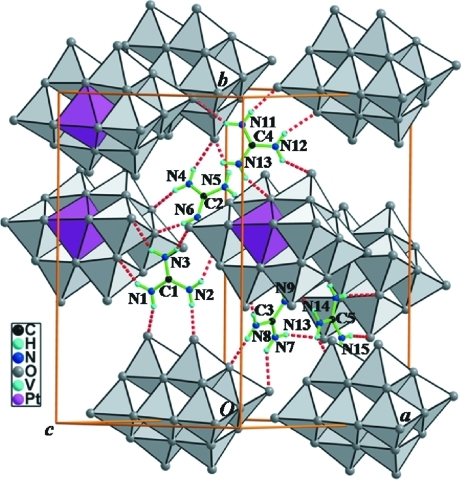
Partial N–H···O hydrogen bond interactions (dotted lines) of guanidinium cations with the O atoms of polyanions.

## supplementary crystallographic information

### Comment

Two heteropolyanions that belong to the decavanadate structure system have recently been reported: the tellurium derivative [HTeV_9_O_28_]^4-^, described by Konaka *et al.* (2011), and the platinum heteropolyoxovanadate, [H_2_PtV_9_O_28_]^5-^, reported by our group in the form of its sodium salt (Lee *et al.*, 2008). The guanidinium ion is a useful  precipitating reagent to enforce separation of polyoxometalates (POMs) species because of the insolubility of its salts in aqueous solution. Since all replaceable counter-cations in POMs can be completely exchanged by guanidinium ions, it is possible to obtain stable POMs by precipitation from aqueous solution with guanidinium salts. We herein report the structure of the title compound as its anhydrous guanidinium salt, obtained by cation exchange from its hydrated sodium salt Na_5_[H_2_PtV_9_O_28_].21H_2_O (Lee *et al.*, 2008).

Fig. 1 shows the structure of the title compound. The geometry of the anion agrees well with that in Na_5_[H_2_PtV_9_O_28_].21H_2_O (Lee *et al.*, 2008). The nine [VO_6_] octahedra in the polyanion are distorted {ranges of V—O (Å): 1.598 (3)–2.395 (2)}, while the [PtO_6_] octahedron is relatively regular {ranges of Pt—O (Å): 1.981 (2)–2.012 (2)}. The two platinum bound µ_2_-O atoms are protonated in the polyanion. These protons are particularly important in the solid state as they lead to the formation of a dimeric assembly, {[H_2_PtV_9_O_28_]_2_}^10-^, through  each of the two µ_2_-O7–H···µ_2_-O19 and µ_2_-O8–H···µ_3_-O4  interanion hydrogen bonds (Fig. 2 & Table 1). The guanidinium cations are hydrogen bonded with µ_2_  and µ_3_-O atoms of the polyanion, with the exceptions of  µ_3_-O5,  µ_2_-O7, µ_2_-O8,  µ_2_-O19,  terminal-O24 and terminal-O27 atoms. The polyanion dimers are connected into a three dimensional network by these hydrogen bonds (Fig. 3 & Table 1).

### Experimental

A pale-brown powder of the title compound was obtained by addition a small excess stoichiometric quantity of guanidinium chloride CH_6_N_3_Cl to a solution of pentasodium nonavanadoplatinate hydrate Na_5_[H_2_PtV_9_O_28_].21H_2_O (Lee *et al.*, 2008). Single crystals were obtained by recrystallization of the crude powder from a boiling aqueous solution.

### Refinement

All H atoms of guanidinium ions were positioned geometrically and refined using a riding model, with N—H = 0.88 Å and *U*_iso_(H) = 1.2*U*_eq_(N). The H7 & H8 atoms bound to µ_2_-O7 and µ_2_-O8, respectively, on the polyanion were found in a difference Fourier map and were refined freely. The unusually short distance of µ_2_-O17···terminal-O21^i^  {2.869 (4) Å, symmetry code as in Fig. 2.} is caused by the neighboring hydrogen bonds between the polyanions of the dimer as shown in Fig. 2. The highest peak in the difference map is 0.85 Å from Pt1 and the largest hole is 0.64 Å from Pt1.

### Crystal data

**Table:**

(CH_6_N_3_)_5_[H_2_PtV_9_O_28_]	*F*(000) = 2704
*M**_r_* = 1404.01	*D*_x_ = 2.567 Mg m^−^^3^
Monoclinic, *P*2_1_/*n*	Mo *K*α radiation, λ = 0.71073 Å
Hall symbol: -P 2yn	Cell parameters from 9860 reflections
*a* = 12.8861 (3) Å	θ = 2.3–28.3°
*b* = 18.5137 (5) Å	µ = 6.15 mm^−^^1^
*c* = 15.2299 (4) Å	*T* = 147 K
β = 91.143 (1)°	Tetragonal prism, dark brown
*V* = 3632.67 (16) Å^3^	0.09 × 0.06 × 0.05 mm
*Z* = 4	

### Data collection

**Table:**

Bruker SMART APEXII CCD diffractometer	9026 independent reflections
Radiation source: rotating anode	7369 reflections with *I* > 2σ(*I*)
graphite multilayer	*R*_int_ = 0.029
Detector resolution: 10.0 pixels mm^-1^	θ_max_ = 28.3°, θ_min_ = 1.7°
φ and ω scans	*h* = −17→17
Absorption correction: multi-scan (*SADABS*; Bruker, 2009)	*k* = −24→20
*T*_min_ = 0.187, *T*_max_ = 0.305	*l* = −20→19
35027 measured reflections	

### Refinement

**Table:**

Refinement on *F*^2^	Primary atom site location: structure-invariant direct methods
Least-squares matrix: full	Secondary atom site location: difference Fourier map
*R*[*F*^2^ > 2σ(*F*^2^)] = 0.029	Hydrogen site location: difference Fourier map
*wR*(*F*^2^) = 0.073	H atoms treated by a mixture of independent and constrained refinement
*S* = 1.04	*w* = 1/[σ^2^(*F*_o_^2^) + (0.0349*P*)^2^ + 5.3131*P*] where *P* = (*F*_o_^2^ + 2*F*_c_^2^)/3
9026 reflections	(Δ/σ)_max_ = 0.002
531 parameters	Δρ_max_ = 2.02 e Å^−^^3^
0 restraints	Δρ_min_ = −0.75 e Å^−^^3^

### Special details

**Table:**

Geometry. All esds (except the esd in the dihedral angle between two l.s. planes) are estimated using the full covariance matrix. The cell esds are taken into account individually in the estimation of esds in distances, angles and torsion angles; correlations between esds in cell parameters are only used when they are defined by crystal symmetry. An approximate (isotropic) treatment of cell esds is used for estimating esds involving l.s. planes.
Refinement. Refinement of F^2^ against ALL reflections. The weighted R-factor wR and goodness of fit S are based on F^2^, conventional R-factors R are based on F, with F set to zero for negative F^2^. The threshold expression of F^2^ > 2sigma(F^2^) is used only for calculating R-factors(gt) etc. and is not relevant to the choice of reflections for refinement. R-factors based on F^2^ are statistically about twice as large as those based on F, and R- factors based on ALL data will be even larger.

### Fractional atomic coordinates and isotropic or equivalent isotropic displacement parameters (Å^2^)

**Table:**

	*x*	*y*	*z*	*U*_iso_*/*U*_eq_	
Pt1	0.542736 (11)	0.577998 (8)	0.369297 (9)	0.01566 (5)	
V1	0.30209 (5)	0.57189 (4)	0.38280 (4)	0.01817 (14)	
V2	0.78344 (5)	0.58912 (4)	0.36257 (4)	0.02039 (14)	
V3	0.77187 (5)	0.53187 (4)	0.16922 (4)	0.02286 (15)	
V4	0.53009 (5)	0.52416 (3)	0.17333 (4)	0.01602 (13)	
V5	0.29080 (5)	0.51648 (4)	0.18896 (4)	0.02100 (14)	
V6	0.40522 (5)	0.66390 (4)	0.23543 (4)	0.01871 (14)	
V7	0.64474 (5)	0.67109 (4)	0.22351 (4)	0.01951 (14)	
V8	0.66979 (5)	0.44125 (3)	0.31842 (4)	0.01746 (14)	
V9	0.42847 (5)	0.43285 (3)	0.32721 (4)	0.01673 (13)	
O1	0.43349 (19)	0.54929 (14)	0.28217 (15)	0.0164 (5)	
O2	0.63984 (19)	0.55714 (13)	0.27333 (16)	0.0162 (5)	
O3	0.53101 (19)	0.67636 (13)	0.31501 (16)	0.0176 (5)	
O4	0.55291 (18)	0.47198 (13)	0.39721 (16)	0.0148 (5)	
O5	0.52143 (19)	0.62722 (14)	0.16275 (15)	0.0179 (5)	
O6	0.54281 (19)	0.43699 (13)	0.23975 (16)	0.0170 (5)	
O7	0.6651 (2)	0.60548 (16)	0.44559 (18)	0.0190 (6)	
H7	0.683 (5)	0.582 (3)	0.481 (4)	0.06 (2)*	
O8	0.4300 (2)	0.59913 (15)	0.45402 (18)	0.0175 (6)	
H8	0.435 (6)	0.572 (4)	0.492 (5)	0.10 (3)*	
O9	0.8507 (2)	0.56308 (15)	0.26057 (18)	0.0238 (6)	
O10	0.6318 (2)	0.51241 (14)	0.10732 (16)	0.0203 (6)	
O11	0.4210 (2)	0.50265 (14)	0.11669 (16)	0.0208 (6)	
O12	0.22238 (19)	0.54062 (14)	0.28785 (17)	0.0200 (6)	
O13	0.32205 (19)	0.66140 (14)	0.33012 (16)	0.0191 (6)	
O14	0.3137 (2)	0.61527 (15)	0.16496 (17)	0.0217 (6)	
O15	0.7300 (2)	0.63003 (15)	0.14740 (17)	0.0228 (6)	
O16	0.7424 (2)	0.67589 (14)	0.31362 (17)	0.0214 (6)	
O17	0.76362 (19)	0.48980 (14)	0.38500 (16)	0.0196 (6)	
O18	0.7499 (2)	0.44365 (15)	0.21814 (17)	0.0228 (6)	
O19	0.34106 (19)	0.47514 (14)	0.40544 (16)	0.0185 (5)	
O20	0.3327 (2)	0.42802 (14)	0.23860 (17)	0.0206 (6)	
O21	0.2156 (2)	0.58921 (15)	0.45354 (18)	0.0246 (6)	
O22	0.8783 (2)	0.61015 (16)	0.42779 (18)	0.0266 (6)	
O23	0.8536 (2)	0.51757 (17)	0.09315 (18)	0.0314 (7)	
O24	0.2018 (2)	0.49432 (17)	0.12069 (19)	0.0316 (7)	
O25	0.3944 (2)	0.74581 (15)	0.20034 (17)	0.0264 (6)	
O26	0.6351 (2)	0.75336 (15)	0.18928 (18)	0.0262 (6)	
O27	0.6792 (2)	0.35837 (15)	0.34966 (18)	0.0246 (6)	
O28	0.4378 (2)	0.35126 (15)	0.36174 (18)	0.0244 (6)	
C1	0.0305 (3)	0.4275 (2)	0.3703 (3)	0.0292 (10)	
N1	−0.0557 (3)	0.3905 (2)	0.3781 (3)	0.0364 (9)	
H1A	−0.0581	0.3449	0.3620	0.044*	
H1B	−0.1112	0.4113	0.3994	0.044*	
N2	0.1144 (3)	0.3970 (2)	0.3384 (3)	0.0491 (12)	
H2A	0.1129	0.3514	0.3221	0.059*	
H2B	0.1718	0.4223	0.3335	0.059*	
N3	0.0348 (3)	0.4953 (2)	0.3973 (3)	0.0426 (10)	
H3A	−0.0201	0.5155	0.4204	0.051*	
H3B	0.0925	0.5202	0.3922	0.051*	
C2	0.0401 (3)	0.6765 (2)	0.1762 (3)	0.0333 (10)	
N4	−0.0499 (3)	0.7121 (2)	0.1651 (3)	0.0395 (10)	
H4A	−0.1081	0.6928	0.1832	0.047*	
H4B	−0.0507	0.7548	0.1397	0.047*	
N5	0.1275 (3)	0.7054 (2)	0.1472 (3)	0.0427 (11)	
H5A	0.1865	0.6819	0.1534	0.051*	
H5B	0.1262	0.7482	0.1219	0.051*	
N6	0.0416 (3)	0.6132 (2)	0.2138 (3)	0.0480 (12)	
H6A	0.1005	0.5896	0.2201	0.058*	
H6B	−0.0164	0.5942	0.2329	0.058*	
C3	0.3111 (4)	0.3016 (2)	0.0616 (3)	0.0318 (10)	
N7	0.2769 (3)	0.2520 (2)	0.0042 (2)	0.0444 (11)	
H7A	0.2482	0.2120	0.0231	0.053*	
H7B	0.2832	0.2595	−0.0526	0.053*	
N8	0.3014 (3)	0.2900 (2)	0.1461 (2)	0.0404 (10)	
H8A	0.3233	0.3225	0.1843	0.048*	
H8B	0.2729	0.2497	0.1645	0.048*	
N9	0.3535 (4)	0.3616 (2)	0.0331 (2)	0.0456 (11)	
H9A	0.3757	0.3945	0.0707	0.055*	
H9B	0.3597	0.3689	−0.0237	0.055*	
C4	0.5247 (4)	0.8411 (3)	0.4233 (3)	0.0425 (12)	
N10	0.4694 (4)	0.7839 (2)	0.4415 (3)	0.0556 (13)	
H10A	0.4826	0.7424	0.4158	0.067*	
H10B	0.4190	0.7869	0.4795	0.067*	
N11	0.5065 (4)	0.9033 (3)	0.4640 (3)	0.0655 (16)	
H11A	0.4573	0.9060	0.5031	0.079*	
H11B	0.5437	0.9418	0.4518	0.079*	
N12	0.6001 (4)	0.8374 (3)	0.3668 (3)	0.0661 (16)	
H12A	0.6139	0.7961	0.3408	0.079*	
H12B	0.6368	0.8761	0.3550	0.079*	
C5	0.6277 (5)	0.3113 (3)	0.0507 (3)	0.0429 (13)	
N13	0.6377 (4)	0.3018 (2)	0.1358 (3)	0.0543 (13)	
H13A	0.6369	0.3392	0.1714	0.065*	
H13B	0.6452	0.2579	0.1573	0.065*	
N14	0.6164 (4)	0.3759 (2)	0.0178 (2)	0.0523 (13)	
H14A	0.6154	0.4138	0.0528	0.063*	
H14B	0.6097	0.3817	−0.0394	0.063*	
N15	0.6325 (5)	0.2548 (2)	−0.0020 (3)	0.085 (2)	
H15A	0.6281	0.2608	−0.0593	0.102*	
H15B	0.6401	0.2112	0.0202	0.102*	

### Atomic displacement parameters (Å^2^)

**Table:**

	*U*^11^	*U*^22^	*U*^33^	*U*^12^	*U*^13^	*U*^23^
Pt1	0.01953 (8)	0.01330 (8)	0.01412 (7)	0.00077 (6)	−0.00017 (5)	0.00008 (6)
V1	0.0179 (3)	0.0203 (4)	0.0163 (3)	0.0029 (3)	−0.0006 (2)	−0.0005 (3)
V2	0.0192 (3)	0.0218 (4)	0.0201 (3)	−0.0011 (3)	−0.0010 (3)	−0.0034 (3)
V3	0.0228 (3)	0.0259 (4)	0.0200 (3)	−0.0002 (3)	0.0048 (3)	−0.0037 (3)
V4	0.0198 (3)	0.0153 (3)	0.0130 (3)	0.0006 (2)	−0.0003 (2)	−0.0014 (2)
V5	0.0207 (3)	0.0236 (4)	0.0186 (3)	0.0002 (3)	−0.0039 (3)	−0.0023 (3)
V6	0.0240 (3)	0.0144 (3)	0.0177 (3)	0.0029 (3)	−0.0027 (3)	0.0025 (3)
V7	0.0247 (3)	0.0151 (3)	0.0188 (3)	−0.0024 (3)	0.0025 (3)	0.0018 (3)
V8	0.0191 (3)	0.0148 (3)	0.0185 (3)	0.0024 (2)	−0.0001 (2)	−0.0004 (2)
V9	0.0192 (3)	0.0124 (3)	0.0186 (3)	−0.0006 (2)	−0.0003 (2)	0.0004 (2)
O1	0.0196 (13)	0.0152 (13)	0.0142 (12)	0.0014 (10)	−0.0025 (10)	−0.0002 (10)
O2	0.0193 (13)	0.0152 (13)	0.0143 (12)	0.0001 (10)	0.0028 (10)	0.0004 (10)
O3	0.0234 (13)	0.0134 (14)	0.0158 (12)	0.0008 (10)	−0.0003 (10)	0.0006 (10)
O4	0.0167 (12)	0.0118 (13)	0.0158 (12)	0.0018 (10)	−0.0018 (10)	0.0007 (10)
O5	0.0251 (14)	0.0149 (14)	0.0136 (12)	0.0003 (11)	−0.0013 (10)	0.0029 (10)
O6	0.0207 (13)	0.0150 (14)	0.0153 (12)	−0.0005 (10)	−0.0024 (10)	−0.0033 (10)
O7	0.0231 (14)	0.0194 (15)	0.0142 (13)	−0.0022 (11)	−0.0032 (11)	−0.0002 (12)
O8	0.0196 (13)	0.0180 (14)	0.0149 (13)	0.0005 (11)	−0.0009 (10)	−0.0001 (11)
O9	0.0218 (14)	0.0248 (16)	0.0249 (14)	−0.0007 (11)	0.0031 (11)	−0.0016 (12)
O10	0.0272 (14)	0.0189 (14)	0.0147 (12)	0.0017 (11)	0.0031 (11)	−0.0012 (11)
O11	0.0281 (14)	0.0189 (15)	0.0154 (13)	0.0003 (11)	−0.0021 (11)	−0.0006 (11)
O12	0.0194 (13)	0.0189 (15)	0.0216 (13)	0.0015 (11)	−0.0032 (11)	0.0003 (11)
O13	0.0225 (13)	0.0164 (14)	0.0182 (13)	0.0054 (11)	−0.0022 (10)	0.0001 (11)
O14	0.0241 (14)	0.0205 (15)	0.0201 (13)	0.0022 (11)	−0.0046 (11)	0.0010 (11)
O15	0.0280 (15)	0.0220 (15)	0.0186 (13)	−0.0042 (12)	0.0044 (11)	−0.0002 (11)
O16	0.0230 (14)	0.0189 (15)	0.0223 (14)	−0.0037 (11)	0.0008 (11)	−0.0016 (11)
O17	0.0194 (13)	0.0204 (15)	0.0189 (13)	0.0016 (11)	−0.0013 (10)	−0.0019 (11)
O18	0.0237 (14)	0.0227 (15)	0.0221 (14)	0.0035 (11)	0.0022 (11)	−0.0043 (12)
O19	0.0202 (13)	0.0168 (14)	0.0183 (13)	0.0008 (10)	0.0002 (10)	0.0017 (11)
O20	0.0227 (14)	0.0187 (15)	0.0204 (13)	−0.0014 (11)	−0.0029 (11)	−0.0007 (11)
O21	0.0217 (14)	0.0289 (17)	0.0235 (14)	0.0026 (12)	0.0033 (11)	−0.0014 (12)
O22	0.0227 (14)	0.0270 (16)	0.0298 (15)	0.0001 (12)	−0.0026 (12)	−0.0061 (13)
O23	0.0305 (16)	0.0401 (19)	0.0238 (15)	−0.0005 (14)	0.0083 (12)	−0.0053 (14)
O24	0.0302 (16)	0.0362 (19)	0.0279 (15)	−0.0004 (14)	−0.0098 (13)	−0.0047 (14)
O25	0.0356 (16)	0.0198 (15)	0.0235 (14)	0.0047 (12)	−0.0037 (12)	0.0046 (12)
O26	0.0352 (16)	0.0154 (15)	0.0280 (15)	−0.0031 (12)	0.0031 (12)	0.0029 (12)
O27	0.0257 (14)	0.0180 (15)	0.0299 (15)	0.0038 (11)	−0.0010 (12)	0.0001 (12)
O28	0.0285 (15)	0.0156 (15)	0.0293 (15)	0.0004 (11)	0.0015 (12)	0.0022 (12)
C1	0.026 (2)	0.022 (2)	0.039 (2)	−0.0003 (18)	−0.0003 (18)	0.0003 (19)
N1	0.0246 (19)	0.027 (2)	0.057 (3)	−0.0022 (16)	−0.0011 (17)	−0.0084 (19)
N2	0.039 (2)	0.021 (2)	0.089 (4)	−0.0033 (18)	0.025 (2)	−0.006 (2)
N3	0.027 (2)	0.023 (2)	0.077 (3)	0.0018 (16)	0.001 (2)	−0.008 (2)
C2	0.023 (2)	0.028 (3)	0.050 (3)	0.0027 (18)	0.001 (2)	0.002 (2)
N4	0.0244 (19)	0.029 (2)	0.065 (3)	0.0039 (16)	0.0051 (18)	0.008 (2)
N5	0.0214 (19)	0.030 (2)	0.077 (3)	0.0020 (16)	0.0051 (19)	0.008 (2)
N6	0.0207 (19)	0.043 (3)	0.080 (3)	0.0048 (18)	0.005 (2)	0.025 (2)
C3	0.049 (3)	0.025 (2)	0.021 (2)	−0.006 (2)	−0.0003 (19)	−0.0023 (18)
N7	0.084 (3)	0.029 (2)	0.0205 (18)	−0.022 (2)	0.0038 (19)	−0.0022 (16)
N8	0.067 (3)	0.034 (2)	0.0209 (18)	−0.024 (2)	0.0022 (18)	−0.0029 (17)
N9	0.088 (3)	0.025 (2)	0.024 (2)	−0.019 (2)	0.005 (2)	−0.0013 (17)
C4	0.059 (3)	0.031 (3)	0.038 (3)	−0.001 (2)	0.001 (2)	−0.008 (2)
N10	0.065 (3)	0.040 (3)	0.062 (3)	−0.014 (2)	0.014 (2)	−0.019 (2)
N11	0.081 (4)	0.036 (3)	0.082 (4)	−0.010 (2)	0.040 (3)	−0.021 (3)
N12	0.094 (4)	0.043 (3)	0.064 (3)	−0.012 (3)	0.039 (3)	−0.024 (2)
C5	0.083 (4)	0.024 (3)	0.022 (2)	0.002 (2)	−0.001 (2)	0.0014 (19)
N13	0.111 (4)	0.027 (2)	0.024 (2)	0.011 (2)	−0.010 (2)	0.0010 (18)
N14	0.120 (4)	0.019 (2)	0.0175 (19)	0.011 (2)	−0.003 (2)	0.0009 (16)
N15	0.211 (7)	0.017 (2)	0.027 (2)	0.014 (3)	−0.008 (3)	0.0019 (19)

### Geometric parameters (Å, °)

**Table:**

Pt1—O2	1.981 (2)	V7—O16	1.846 (3)
Pt1—O1	1.988 (2)	V7—O5	1.995 (3)
Pt1—O8	2.001 (3)	V7—O3	2.045 (2)
Pt1—O3	2.004 (2)	V7—O2	2.243 (3)
Pt1—O7	2.005 (3)	V8—O27	1.610 (3)
Pt1—O4	2.012 (2)	V8—O17	1.803 (3)
Pt1—V6	3.1116 (6)	V8—O18	1.861 (3)
Pt1—V2	3.1122 (7)	V8—O6	2.010 (3)
Pt1—V1	3.1139 (6)	V8—O4	2.026 (2)
Pt1—V8	3.1212 (6)	V8—O2	2.283 (3)
Pt1—V7	3.1216 (6)	V8—V9	3.1192 (9)
Pt1—V9	3.1245 (6)	V9—O28	1.603 (3)
V1—O21	1.598 (3)	V9—O20	1.813 (3)
V1—O12	1.850 (3)	V9—O19	1.832 (2)
V1—O13	1.861 (3)	V9—O6	2.007 (2)
V1—O19	1.890 (3)	V9—O4	2.041 (3)
V1—O8	2.019 (3)	V9—O1	2.263 (3)
V1—O1	2.344 (2)	O7—H7	0.73 (6)
V1—V5	3.1259 (9)	O8—H8	0.77 (7)
V1—V6	3.1343 (9)	O17—O21^i^	2.869 (4)
V1—V9	3.1701 (9)	C1—N1	1.313 (5)
V2—O22	1.608 (3)	C1—N2	1.321 (5)
V2—O16	1.844 (3)	C1—N3	1.321 (6)
V2—O9	1.857 (3)	N1—H1A	0.8800
V2—O17	1.888 (3)	N1—H1B	0.8800
V2—O7	2.024 (3)	N2—H2A	0.8800
V2—O2	2.350 (3)	N2—H2B	0.8800
V2—V3	3.1303 (9)	N3—H3A	0.8800
V2—V7	3.1355 (10)	N3—H3B	0.8800
V2—V8	3.1705 (9)	C2—N6	1.303 (6)
V3—O23	1.603 (3)	C2—N5	1.330 (5)
V3—O9	1.801 (3)	C2—N4	1.342 (5)
V3—O18	1.820 (3)	N4—H4A	0.8800
V3—O15	1.923 (3)	N4—H4B	0.8800
V3—O10	2.051 (3)	N5—H5A	0.8800
V3—O2	2.395 (2)	N5—H5B	0.8800
V3—V4	3.1207 (9)	N6—H6A	0.8800
V3—V8	3.1349 (9)	N6—H6B	0.8800
V3—V7	3.1724 (9)	C3—N8	1.313 (5)
V4—O10	1.682 (2)	C3—N9	1.316 (5)
V4—O11	1.683 (3)	C3—N7	1.337 (5)
V4—O6	1.910 (3)	N7—H7A	0.8800
V4—O5	1.918 (3)	N7—H7B	0.8800
V4—O1	2.144 (2)	N8—H8A	0.8800
V4—O2	2.146 (3)	N8—H8B	0.8800
V4—V5	3.1007 (9)	N9—H9A	0.8800
V4—V7	3.1813 (9)	N9—H9B	0.8800
V4—V9	3.1917 (8)	C4—N10	1.310 (6)
V4—V8	3.2127 (9)	C4—N12	1.312 (6)
V5—O24	1.586 (3)	C4—N11	1.331 (6)
V5—O12	1.816 (3)	N10—H10A	0.8800
V5—O20	1.878 (3)	N10—H10B	0.8800
V5—O14	1.890 (3)	N11—H11A	0.8800
V5—O11	2.041 (3)	N11—H11B	0.8800
V5—O1	2.379 (3)	N12—H12A	0.8800
V5—V9	3.1350 (9)	N12—H12B	0.8800
V5—V6	3.1752 (10)	C5—N14	1.304 (6)
V6—O25	1.613 (3)	C5—N13	1.313 (6)
V6—O13	1.814 (2)	C5—N15	1.321 (6)
V6—O14	1.817 (3)	N13—H13A	0.8800
V6—O5	1.999 (2)	N13—H13B	0.8800
V6—O3	2.018 (3)	N14—H14A	0.8800
V6—O1	2.265 (3)	N14—H14B	0.8800
V6—V7	3.0983 (9)	N15—H15A	0.8800
V7—O26	1.614 (3)	N15—H15B	0.8800
V7—O15	1.783 (3)		
			
O2—Pt1—O1	84.53 (10)	O24—V5—Pt1	178.05 (11)
O2—Pt1—O8	172.49 (11)	O12—V5—Pt1	77.36 (8)
O1—Pt1—O8	88.24 (10)	O20—V5—Pt1	76.70 (8)
O2—Pt1—O3	85.20 (10)	O14—V5—Pt1	75.62 (8)
O1—Pt1—O3	85.47 (10)	O11—V5—Pt1	76.28 (7)
O8—Pt1—O3	92.21 (11)	O1—V5—Pt1	2.55 (6)
O2—Pt1—O7	88.64 (11)	V4—V5—Pt1	46.173 (14)
O1—Pt1—O7	173.15 (10)	V1—V5—Pt1	45.540 (14)
O8—Pt1—O7	98.57 (11)	V9—V5—Pt1	45.729 (14)
O3—Pt1—O7	93.47 (11)	V6—V5—Pt1	45.470 (14)
O2—Pt1—O4	85.77 (10)	O25—V6—O13	103.81 (13)
O1—Pt1—O4	85.55 (10)	O25—V6—O14	102.67 (14)
O8—Pt1—O4	95.72 (10)	O13—V6—O14	94.04 (12)
O3—Pt1—O4	167.82 (10)	O25—V6—O5	101.34 (12)
O7—Pt1—O4	94.47 (11)	O13—V6—O5	153.19 (11)
O2—Pt1—V6	88.63 (7)	O14—V6—O5	89.37 (11)
O1—Pt1—V6	46.58 (7)	O25—V6—O3	98.90 (13)
O8—Pt1—V6	84.91 (8)	O13—V6—O3	90.32 (11)
O3—Pt1—V6	39.48 (7)	O14—V6—O3	156.26 (12)
O7—Pt1—V6	132.91 (9)	O5—V6—O3	76.68 (10)
O4—Pt1—V6	132.13 (7)	O25—V6—O1	175.61 (12)
O2—Pt1—V2	49.00 (8)	O13—V6—O1	79.64 (10)
O1—Pt1—V2	133.53 (7)	O14—V6—O1	79.62 (11)
O8—Pt1—V2	138.17 (8)	O5—V6—O1	74.83 (9)
O3—Pt1—V2	89.62 (7)	O3—V6—O1	78.23 (10)
O7—Pt1—V2	39.63 (8)	O25—V6—V7	91.14 (11)
O4—Pt1—V2	90.61 (7)	O13—V6—V7	130.73 (9)
V6—Pt1—V2	119.971 (17)	O14—V6—V7	128.46 (9)
O2—Pt1—V1	133.33 (8)	O5—V6—V7	39.09 (7)
O1—Pt1—V1	48.80 (7)	O3—V6—V7	40.62 (7)
O8—Pt1—V1	39.44 (8)	O1—V6—V7	84.52 (6)
O3—Pt1—V1	89.61 (7)	O25—V6—Pt1	137.90 (11)
O7—Pt1—V1	138.01 (8)	O13—V6—Pt1	78.69 (8)
O4—Pt1—V1	90.65 (7)	O14—V6—Pt1	119.21 (9)
V6—Pt1—V1	60.459 (17)	O5—V6—Pt1	76.47 (7)
V2—Pt1—V1	177.448 (17)	O3—V6—Pt1	39.16 (7)
O2—Pt1—V8	46.88 (7)	O1—V6—Pt1	39.60 (6)
O1—Pt1—V8	89.16 (7)	V7—V6—Pt1	60.354 (17)
O8—Pt1—V8	135.23 (8)	O25—V6—V1	135.49 (10)
O3—Pt1—V8	132.08 (7)	O13—V6—V1	31.94 (8)
O7—Pt1—V8	86.53 (8)	O14—V6—V1	82.78 (9)
O4—Pt1—V8	39.54 (7)	O5—V6—V1	123.02 (8)
V6—Pt1—V8	123.136 (17)	O3—V6—V1	88.80 (7)
V2—Pt1—V8	61.145 (17)	O1—V6—V1	48.22 (6)
V1—Pt1—V8	120.993 (17)	V7—V6—V1	120.16 (3)
O2—Pt1—V7	45.71 (7)	Pt1—V6—V1	59.808 (16)
O1—Pt1—V7	88.59 (7)	O25—V6—V5	134.23 (11)
O8—Pt1—V7	132.24 (8)	O13—V6—V5	82.89 (9)
O3—Pt1—V7	40.03 (7)	O14—V6—V5	31.74 (9)
O7—Pt1—V7	86.26 (8)	O5—V6—V5	86.23 (8)
O4—Pt1—V7	131.48 (7)	O3—V6—V5	126.59 (8)
V6—Pt1—V7	59.613 (17)	O1—V6—V5	48.39 (6)
V2—Pt1—V7	60.396 (18)	V7—V6—V5	118.85 (3)
V1—Pt1—V7	120.068 (17)	Pt1—V6—V5	87.86 (2)
V8—Pt1—V7	92.341 (17)	V1—V6—V5	59.39 (2)
O2—Pt1—V9	89.03 (7)	O26—V7—O15	103.70 (13)
O1—Pt1—V9	46.24 (7)	O26—V7—O16	103.99 (14)
O8—Pt1—V9	87.44 (8)	O15—V7—O16	94.81 (12)
O3—Pt1—V9	131.71 (7)	O26—V7—O5	100.30 (13)
O7—Pt1—V9	134.34 (9)	O15—V7—O5	91.17 (11)
O4—Pt1—V9	39.90 (7)	O16—V7—O5	152.79 (11)
V6—Pt1—V9	92.592 (17)	O26—V7—O3	97.09 (12)
V2—Pt1—V9	121.067 (17)	O15—V7—O3	157.33 (12)
V1—Pt1—V9	61.082 (17)	O16—V7—O3	88.77 (11)
V8—Pt1—V9	59.923 (16)	O5—V7—O3	76.14 (10)
V7—Pt1—V9	122.162 (17)	O26—V7—O2	173.88 (12)
O21—V1—O12	101.90 (13)	O15—V7—O2	80.84 (11)
O21—V1—O13	102.48 (13)	O16—V7—O2	79.47 (11)
O12—V1—O13	91.21 (12)	O5—V7—O2	75.32 (10)
O21—V1—O19	104.68 (13)	O3—V7—O2	77.81 (9)
O12—V1—O19	89.28 (12)	O26—V7—V6	89.37 (10)
O13—V1—O19	152.12 (11)	O15—V7—V6	130.34 (9)
O21—V1—O8	99.23 (13)	O16—V7—V6	128.61 (8)
O12—V1—O8	158.87 (11)	O5—V7—V6	39.17 (7)
O13—V1—O8	83.73 (12)	O3—V7—V6	39.98 (7)
O19—V1—O8	85.85 (12)	O2—V7—V6	84.55 (7)
O21—V1—O1	177.76 (13)	O26—V7—Pt1	136.05 (10)
O12—V1—O1	80.22 (10)	O15—V7—Pt1	120.04 (9)
O13—V1—O1	76.66 (10)	O16—V7—Pt1	77.78 (8)
O19—V1—O1	75.96 (10)	O5—V7—Pt1	76.26 (7)
O8—V1—O1	78.65 (10)	O3—V7—Pt1	39.09 (7)
O21—V1—Pt1	138.24 (11)	O2—V7—Pt1	39.20 (6)
O12—V1—Pt1	119.86 (8)	V6—V7—Pt1	60.033 (16)
O13—V1—Pt1	78.04 (8)	O26—V7—V2	135.58 (11)
O19—V1—Pt1	77.59 (8)	O15—V7—V2	83.25 (9)
O8—V1—Pt1	39.01 (7)	O16—V7—V2	31.79 (8)
O1—V1—Pt1	39.65 (6)	O5—V7—V2	123.65 (8)
O21—V1—V5	133.06 (11)	O3—V7—V2	88.25 (7)
O12—V1—V5	31.18 (8)	O2—V7—V2	48.39 (7)
O13—V1—V5	83.65 (8)	V6—V7—V2	119.65 (2)
O19—V1—V5	82.44 (8)	Pt1—V7—V2	59.655 (17)
O8—V1—V5	127.69 (8)	O26—V7—V3	136.01 (10)
O1—V1—V5	49.05 (6)	O15—V7—V3	32.45 (9)
Pt1—V1—V5	88.695 (19)	O16—V7—V3	83.39 (8)
O21—V1—V6	133.24 (11)	O5—V7—V3	87.65 (8)
O12—V1—V6	81.20 (8)	O3—V7—V3	126.67 (8)
O13—V1—V6	31.05 (8)	O2—V7—V3	48.87 (6)
O19—V1—V6	122.06 (8)	V6—V7—V3	120.04 (3)
O8—V1—V6	84.01 (8)	Pt1—V7—V3	87.92 (2)
O1—V1—V6	46.11 (6)	V2—V7—V3	59.50 (2)
Pt1—V1—V6	59.733 (16)	O26—V7—V4	134.18 (11)
V5—V1—V6	60.96 (2)	O15—V7—V4	76.62 (9)
O21—V1—V9	135.39 (11)	O16—V7—V4	121.76 (9)
O12—V1—V9	79.48 (8)	O5—V7—V4	34.81 (7)
O13—V1—V9	122.12 (8)	O3—V7—V4	82.46 (7)
O19—V1—V9	31.04 (7)	O2—V7—V4	42.36 (7)
O8—V1—V9	85.87 (8)	V6—V7—V4	61.25 (2)
O1—V1—V9	45.48 (6)	Pt1—V7—V4	59.913 (16)
Pt1—V1—V9	59.623 (16)	V2—V7—V4	90.24 (2)
V5—V1—V9	59.72 (2)	V3—V7—V4	58.83 (2)
V6—V1—V9	91.30 (2)	O27—V8—O17	105.25 (13)
O21—V1—V4	176.81 (11)	O27—V8—O18	103.02 (13)
O12—V1—V4	75.10 (8)	O17—V8—O18	94.17 (12)
O13—V1—V4	76.69 (8)	O27—V8—O6	101.22 (13)
O19—V1—V4	76.51 (7)	O17—V8—O6	152.09 (11)
O8—V1—V4	83.77 (7)	O18—V8—O6	88.30 (11)
O1—V1—V4	5.14 (6)	O27—V8—O4	98.35 (12)
Pt1—V1—V4	44.779 (11)	O17—V8—O4	91.43 (11)
V5—V1—V4	43.920 (16)	O18—V8—O4	155.59 (12)
V6—V1—V4	45.732 (16)	O6—V8—O4	76.00 (10)
V9—V1—V4	45.584 (16)	O27—V8—O2	174.58 (12)
O22—V2—O16	104.30 (14)	O17—V8—O2	78.93 (11)
O22—V2—O9	102.60 (13)	O18—V8—O2	79.87 (11)
O16—V2—O9	91.29 (12)	O6—V8—O2	74.15 (10)
O22—V2—O17	103.18 (14)	O4—V8—O2	77.94 (9)
O16—V2—O17	151.97 (12)	O27—V8—V9	90.54 (10)
O9—V2—O17	88.03 (12)	O17—V8—V9	131.21 (8)
O22—V2—O7	98.69 (13)	O18—V8—V9	127.33 (9)
O16—V2—O7	84.76 (12)	O6—V8—V9	39.03 (7)
O9—V2—O7	158.66 (12)	O4—V8—V9	40.09 (7)
O17—V2—O7	85.78 (11)	O2—V8—V9	84.09 (6)
O22—V2—O2	177.18 (12)	O27—V8—Pt1	137.43 (10)
O16—V2—O2	76.68 (10)	O17—V8—Pt1	78.76 (8)
O9—V2—O2	79.97 (11)	O18—V8—Pt1	119.13 (9)
O17—V2—O2	75.62 (10)	O6—V8—Pt1	75.82 (7)
O7—V2—O2	78.71 (10)	O4—V8—Pt1	39.22 (7)
O22—V2—Pt1	137.88 (10)	O2—V8—Pt1	39.29 (6)
O16—V2—Pt1	78.07 (8)	V9—V8—Pt1	60.091 (16)
O9—V2—Pt1	119.48 (9)	O27—V8—V3	133.93 (10)
O17—V2—Pt1	77.95 (8)	O17—V8—V3	81.63 (8)
O7—V2—Pt1	39.20 (8)	O18—V8—V3	31.21 (9)
O2—V2—Pt1	39.51 (6)	O6—V8—V3	86.41 (7)
O22—V2—V3	133.20 (10)	O4—V8—V3	127.35 (8)
O16—V2—V3	84.65 (8)	O2—V8—V3	49.46 (6)
O9—V2—V3	30.64 (9)	V9—V8—V3	119.40 (3)
O17—V2—V3	80.59 (8)	Pt1—V8—V3	88.60 (2)
O7—V2—V3	128.03 (8)	O27—V8—V2	136.74 (10)
O2—V2—V3	49.34 (6)	O17—V8—V2	31.60 (8)
Pt1—V2—V3	88.84 (2)	O18—V8—V2	83.79 (9)
O22—V2—V7	135.75 (11)	O6—V8—V2	121.82 (8)
O16—V2—V7	31.83 (8)	O4—V8—V2	88.72 (7)
O9—V2—V7	80.20 (9)	O2—V8—V2	47.70 (7)
O17—V2—V7	121.07 (8)	V9—V8—V2	119.38 (2)
O7—V2—V7	85.58 (9)	Pt1—V8—V2	59.289 (17)
O2—V2—V7	45.54 (6)	V3—V8—V2	59.53 (2)
Pt1—V2—V7	59.949 (17)	O27—V8—V4	134.15 (10)
V3—V2—V7	60.84 (2)	O17—V8—V4	120.60 (9)
O22—V2—V8	133.04 (11)	O18—V8—V4	74.73 (9)
O16—V2—V8	122.62 (9)	O6—V8—V4	33.95 (7)
O9—V2—V8	79.61 (9)	O4—V8—V4	81.96 (7)
O17—V2—V8	30.01 (8)	O2—V8—V4	41.85 (7)
O7—V2—V8	84.89 (8)	V9—V8—V4	60.519 (19)
O2—V2—V8	45.95 (6)	Pt1—V8—V4	59.579 (16)
Pt1—V2—V8	59.566 (16)	V3—V8—V4	58.88 (2)
V3—V2—V8	59.67 (2)	V2—V8—V4	89.05 (2)
V7—V2—V8	91.15 (2)	O28—V9—O20	104.09 (13)
O22—V2—V4	177.21 (10)	O28—V9—O19	103.45 (13)
O16—V2—V4	76.96 (8)	O20—V9—O19	94.99 (12)
O9—V2—V4	74.81 (9)	O28—V9—O6	101.66 (12)
O17—V2—V4	75.84 (8)	O20—V9—O6	90.33 (11)
O7—V2—V4	83.87 (8)	O19—V9—O6	152.18 (11)
O2—V2—V4	5.16 (6)	O28—V9—O4	96.30 (13)
Pt1—V2—V4	44.673 (12)	O20—V9—O4	157.32 (11)
V3—V2—V4	44.184 (16)	O19—V9—O4	89.74 (11)
V7—V2—V4	45.295 (17)	O6—V9—O4	75.73 (10)
V8—V2—V4	45.861 (16)	O28—V9—O1	173.88 (12)
O23—V3—O9	104.06 (14)	O20—V9—O1	81.06 (10)
O23—V3—O18	104.91 (14)	O19—V9—O1	79.15 (10)
O9—V3—O18	93.52 (13)	O6—V9—O1	74.74 (10)
O23—V3—O15	102.55 (14)	O4—V9—O1	78.08 (9)
O9—V3—O15	88.98 (12)	O28—V9—V8	89.57 (10)
O18—V3—O15	150.91 (11)	O20—V9—V8	129.43 (9)
O23—V3—O10	102.92 (13)	O19—V9—V8	129.28 (9)
O9—V3—O10	152.70 (11)	O6—V9—V8	39.10 (7)
O18—V3—O10	83.57 (12)	O4—V9—V8	39.74 (7)
O15—V3—O10	81.10 (11)	O1—V9—V8	84.55 (6)
O23—V3—O2	175.10 (13)	O28—V9—Pt1	135.31 (11)
O9—V3—O2	79.80 (10)	O20—V9—Pt1	120.42 (9)
O18—V3—O2	77.65 (10)	O19—V9—Pt1	78.01 (8)
O15—V3—O2	74.29 (10)	O6—V9—Pt1	75.77 (7)
O10—V3—O2	73.05 (9)	O4—V9—Pt1	39.23 (7)
O23—V3—V4	132.64 (11)	O1—V9—Pt1	39.37 (6)
O9—V3—V4	123.15 (9)	V8—V9—Pt1	59.986 (16)
O18—V3—V4	77.73 (9)	O28—V9—V5	136.34 (11)
O15—V3—V4	76.70 (8)	O20—V9—V5	32.50 (8)
O10—V3—V4	29.73 (7)	O19—V9—V5	83.03 (8)
O2—V3—V4	43.36 (6)	O6—V9—V5	87.05 (8)
O23—V3—V2	135.73 (11)	O4—V9—V5	127.14 (7)
O9—V3—V2	31.70 (8)	O1—V9—V5	49.11 (6)
O18—V3—V2	85.62 (9)	V8—V9—V5	119.86 (2)
O15—V3—V2	81.40 (8)	Pt1—V9—V5	88.34 (2)
O10—V3—V2	121.11 (7)	O28—V9—V1	135.49 (10)
O2—V3—V2	48.10 (6)	O20—V9—V1	83.87 (8)
V4—V3—V2	91.46 (2)	O19—V9—V1	32.15 (8)
O23—V3—V8	136.60 (12)	O6—V9—V1	122.28 (8)
O9—V3—V8	81.38 (9)	O4—V9—V1	88.55 (7)
O18—V3—V8	32.00 (8)	O1—V9—V1	47.59 (6)
O15—V3—V8	120.72 (8)	V8—V9—V1	119.27 (3)
O10—V3—V8	82.01 (7)	Pt1—V9—V1	59.295 (16)
O2—V3—V8	46.43 (6)	V5—V9—V1	59.44 (2)
V4—V3—V8	61.80 (2)	O28—V9—V4	135.28 (10)
V2—V3—V8	60.80 (2)	O20—V9—V4	76.28 (8)
O23—V3—V7	132.27 (12)	O19—V9—V4	121.19 (8)
O9—V3—V7	79.87 (9)	O6—V9—V4	34.44 (7)
O18—V3—V7	122.50 (8)	O4—V9—V4	82.30 (7)
O15—V3—V7	29.84 (7)	O1—V9—V4	42.14 (6)
O10—V3—V7	78.95 (7)	V8—V9—V4	61.191 (19)
O2—V3—V7	44.88 (6)	Pt1—V9—V4	59.772 (16)
V4—V3—V7	60.73 (2)	V5—V9—V4	58.686 (19)
V2—V3—V7	59.66 (2)	V1—V9—V4	89.23 (2)
V8—V3—V7	91.12 (2)	Pt1—O1—V4	99.19 (10)
O23—V3—Pt1	177.51 (12)	Pt1—O1—V9	94.39 (10)
O9—V3—Pt1	77.11 (8)	V4—O1—V9	92.76 (10)
O18—V3—Pt1	77.13 (8)	Pt1—O1—V6	93.82 (10)
O15—V3—Pt1	75.21 (7)	V4—O1—V6	93.01 (9)
O10—V3—Pt1	75.77 (7)	V9—O1—V6	169.09 (12)
O2—V3—Pt1	2.81 (6)	Pt1—O1—V1	91.55 (9)
V4—V3—Pt1	46.065 (13)	V4—O1—V1	169.24 (13)
V2—V3—Pt1	45.410 (14)	V9—O1—V1	86.93 (8)
V8—V3—Pt1	45.571 (14)	V6—O1—V1	85.67 (9)
V7—V3—Pt1	45.558 (13)	Pt1—O1—V5	174.41 (12)
O10—V4—O11	108.43 (13)	V4—O1—V5	86.39 (9)
O10—V4—O6	98.49 (12)	V9—O1—V5	84.91 (9)
O11—V4—O6	97.67 (12)	V6—O1—V5	86.22 (9)
O10—V4—O5	97.08 (12)	V1—O1—V5	82.87 (8)
O11—V4—O5	98.38 (12)	Pt1—O2—V4	99.33 (10)
O6—V4—O5	152.79 (11)	Pt1—O2—V7	95.09 (10)
O10—V4—O1	164.10 (12)	V4—O2—V7	92.87 (10)
O11—V4—O1	87.45 (11)	Pt1—O2—V8	93.83 (9)
O6—V4—O1	79.56 (10)	V4—O2—V8	92.93 (10)
O5—V4—O1	79.36 (10)	V7—O2—V8	168.43 (12)
O10—V4—O2	87.17 (11)	Pt1—O2—V2	91.48 (10)
O11—V4—O2	164.40 (11)	V4—O2—V2	169.19 (12)
O6—V4—O2	79.41 (10)	V7—O2—V2	86.07 (9)
O5—V4—O2	79.22 (10)	V8—O2—V2	86.35 (9)
O1—V4—O2	76.95 (9)	Pt1—O2—V3	173.80 (14)
O10—V4—V5	145.90 (9)	V4—O2—V3	86.64 (9)
O11—V4—V5	37.48 (9)	V7—O2—V3	86.25 (8)
O6—V4—V5	89.74 (8)	V8—O2—V3	84.11 (8)
O5—V4—V5	89.76 (8)	V2—O2—V3	82.56 (8)
O1—V4—V5	49.98 (7)	Pt1—O3—V6	101.36 (11)
O2—V4—V5	126.92 (7)	Pt1—O3—V7	100.87 (11)
O10—V4—V3	37.22 (9)	V6—O3—V7	99.40 (11)
O11—V4—V3	145.58 (9)	Pt1—O4—V8	101.24 (11)
O6—V4—V3	88.52 (8)	Pt1—O4—V9	100.88 (11)
O5—V4—V3	90.53 (8)	V8—O4—V9	100.17 (11)
O1—V4—V3	126.93 (7)	V4—O5—V7	108.75 (12)
O2—V4—V3	50.00 (6)	V4—O5—V6	109.54 (12)
V5—V4—V3	176.75 (3)	V7—O5—V6	101.74 (11)
O10—V4—Pt1	125.56 (9)	V4—O6—V9	109.10 (12)
O11—V4—Pt1	126.01 (9)	V4—O6—V8	110.04 (12)
O6—V4—Pt1	76.30 (7)	V9—O6—V8	101.86 (11)
O5—V4—Pt1	76.49 (7)	Pt1—O7—V2	101.17 (12)
O1—V4—Pt1	38.56 (7)	Pt1—O7—H7	121 (5)
O2—V4—Pt1	38.39 (6)	V2—O7—H7	99 (5)
V5—V4—Pt1	88.538 (19)	Pt1—O8—V1	101.55 (12)
V3—V4—Pt1	88.38 (2)	Pt1—O8—H8	108 (6)
O10—V4—V7	83.71 (9)	V1—O8—H8	107 (6)
O11—V4—V7	134.82 (10)	V3—O9—V2	117.66 (14)
O6—V4—V7	124.10 (8)	V4—O10—V3	113.05 (13)
O5—V4—V7	36.44 (8)	V4—O11—V5	112.42 (13)
O1—V4—V7	84.42 (7)	V5—O12—V1	117.00 (13)
O2—V4—V7	44.77 (7)	V6—O13—V1	117.01 (13)
V5—V4—V7	118.59 (3)	V6—O14—V5	117.87 (14)
V3—V4—V7	60.44 (2)	V7—O15—V3	117.71 (13)
Pt1—V4—V7	59.101 (16)	V2—O16—V7	116.38 (14)
O10—V4—V9	134.95 (9)	V8—O17—V2	118.38 (14)
O11—V4—V9	84.27 (9)	V8—O17—O21^i^	106.13 (12)
O6—V4—V9	36.46 (7)	V2—O17—O21^i^	129.86 (13)
O5—V4—V9	124.37 (7)	V3—O18—V8	116.79 (14)
O1—V4—V9	45.10 (7)	V9—O19—V1	116.81 (13)
O2—V4—V9	84.48 (7)	V9—O20—V5	116.27 (14)
V5—V4—V9	59.74 (2)	N1—C1—N2	120.7 (4)
V3—V4—V9	117.60 (3)	N1—C1—N3	119.8 (4)
Pt1—V4—V9	59.053 (16)	N2—C1—N3	119.4 (4)
V7—V4—V9	118.15 (2)	C1—N1—H1A	120.0
O10—V4—V8	85.22 (9)	C1—N1—H1B	120.0
O11—V4—V8	133.67 (9)	H1A—N1—H1B	120.0
O6—V4—V8	36.00 (8)	C1—N2—H2A	120.0
O5—V4—V8	124.33 (8)	C1—N2—H2B	120.0
O1—V4—V8	84.14 (7)	H2A—N2—H2B	120.0
O2—V4—V8	45.22 (7)	C1—N3—H3A	120.0
V5—V4—V8	118.02 (2)	C1—N3—H3B	120.0
V3—V4—V8	59.32 (2)	H3A—N3—H3B	120.0
Pt1—V4—V8	58.765 (16)	N6—C2—N5	120.2 (4)
V7—V4—V8	89.55 (2)	N6—C2—N4	120.1 (4)
V9—V4—V8	58.291 (19)	N5—C2—N4	119.7 (4)
O24—V5—O12	104.52 (14)	C2—N4—H4A	120.0
O24—V5—O20	103.68 (14)	C2—N4—H4B	120.0
O12—V5—O20	91.21 (12)	H4A—N4—H4B	120.0
O24—V5—O14	103.73 (14)	C2—N5—H5A	120.0
O12—V5—O14	90.15 (12)	C2—N5—H5B	120.0
O20—V5—O14	151.27 (12)	H5A—N5—H5B	120.0
O24—V5—O11	101.84 (13)	C2—N6—H6A	120.0
O12—V5—O11	153.64 (11)	C2—N6—H6B	120.0
O20—V5—O11	82.82 (11)	H6A—N6—H6B	120.0
O14—V5—O11	83.32 (11)	N8—C3—N9	120.7 (4)
O24—V5—O1	175.53 (13)	N8—C3—N7	119.5 (4)
O12—V5—O1	79.90 (10)	N9—C3—N7	119.8 (4)
O20—V5—O1	76.70 (10)	C3—N7—H7A	120.0
O14—V5—O1	75.30 (10)	C3—N7—H7B	120.0
O11—V5—O1	73.74 (9)	H7A—N7—H7B	120.0
O24—V5—V4	131.95 (11)	C3—N8—H8A	120.0
O12—V5—V4	123.53 (9)	C3—N8—H8B	120.0
O20—V5—V4	78.03 (8)	H8A—N8—H8B	120.0
O14—V5—V4	77.35 (8)	C3—N9—H9A	120.0
O11—V5—V4	30.11 (7)	C3—N9—H9B	120.0
O1—V5—V4	43.63 (6)	H9A—N9—H9B	120.0
O24—V5—V1	136.33 (11)	N10—C4—N12	120.6 (5)
O12—V5—V1	31.82 (8)	N10—C4—N11	119.9 (5)
O20—V5—V1	84.17 (8)	N12—C4—N11	119.4 (5)
O14—V5—V1	81.98 (8)	C4—N10—H10A	120.0
O11—V5—V1	121.82 (8)	C4—N10—H10B	120.0
O1—V5—V1	48.08 (6)	H10A—N10—H10B	120.0
V4—V5—V1	91.71 (2)	C4—N11—H11A	120.0
O24—V5—V9	134.78 (12)	C4—N11—H11B	120.0
O12—V5—V9	80.92 (8)	H11A—N11—H11B	120.0
O20—V5—V9	31.23 (8)	C4—N12—H12A	120.0
O14—V5—V9	121.29 (8)	C4—N12—H12B	120.0
O11—V5—V9	80.79 (8)	H12A—N12—H12B	120.0
O1—V5—V9	45.98 (6)	N14—C5—N13	120.8 (4)
V4—V5—V9	61.57 (2)	N14—C5—N15	120.0 (4)
V1—V5—V9	60.84 (2)	N13—C5—N15	119.2 (4)
O24—V5—V6	134.00 (12)	C5—N13—H13A	120.0
O12—V5—V6	80.48 (9)	C5—N13—H13B	120.0
O20—V5—V6	122.09 (8)	H13A—N13—H13B	120.0
O14—V5—V6	30.39 (8)	C5—N14—H14A	120.0
O11—V5—V6	81.08 (8)	C5—N14—H14B	120.0
O1—V5—V6	45.39 (6)	H14A—N14—H14B	120.0
V4—V5—V6	61.29 (2)	C5—N15—H15A	120.0
V1—V5—V6	59.65 (2)	C5—N15—H15B	120.0
V9—V5—V6	91.19 (2)	H15A—N15—H15B	120.0

Symmetry codes: (i) −*x*+1, −*y*+1, −*z*+1.

### Hydrogen-bond geometry (Å, °)

**Table:**

*D*—H···*A*	*D*—H	H···*A*	*D*···*A*	*D*—H···*A*
O7—H7···O19^i^	0.73 (6)	2.06 (6)	2.718 (4)	152 (7)
O8—H8···O4^i^	0.77 (7)	1.87 (8)	2.626 (4)	165 (8)
N1—H1A···O26^ii^	0.88	2.11	2.916 (5)	153.
N1—H1B···O17^iii^	0.88	2.18	2.970 (5)	149.
N2—H2A···O25^ii^	0.88	1.99	2.863 (5)	173.
N2—H2B···O12	0.88	2.39	3.105 (5)	138.
N3—H3A···O22^iii^	0.88	2.19	2.973 (5)	148.
N3—H3B···O21	0.88	2.23	3.018 (5)	149.
N4—H4A···O15^iii^	0.88	2.44	3.224 (5)	149.
N4—H4B···O28^iv^	0.88	2.30	2.985 (5)	134.
N5—H5A···O14	0.88	2.06	2.932 (5)	173.
N5—H5B···O28^iv^	0.88	2.10	2.830 (5)	140.
N6—H6A···O12	0.88	2.07	2.899 (5)	156.
N6—H6B···O9^iii^	0.88	1.86	2.737 (5)	171.
N7—H7A···O21^ii^	0.88	2.35	3.084 (5)	142.
N7—H7B···O26^v^	0.88	2.36	3.179 (5)	154.
N8—H8A···O20	0.88	2.12	2.942 (5)	154.
N8—H8B···O13^ii^	0.88	2.04	2.890 (4)	161.
N9—H9A···O11	0.88	2.20	3.025 (5)	157.
N9—H9B···O15^v^	0.88	2.19	2.936 (5)	142.
N10—H10A···O3	0.88	2.07	2.892 (5)	156.
N10—H10B···N7^iv^	0.88	2.62	3.349 (6)	141.
N11—H11A···O23^vi^	0.88	2.40	3.171 (5)	147.
N11—H11B···O23^vii^	0.88	2.06	2.923 (6)	168.
N12—H12A···O26	0.88	2.46	3.159 (5)	137.
N12—H12B···O18^vii^	0.88	2.24	3.063 (5)	157.
N13—H13A···O6	0.88	2.42	3.216 (5)	150.
N13—H13B···O16^viii^	0.88	2.14	2.892 (5)	143.
N14—H14A···O10	0.88	2.02	2.876 (5)	165.
N14—H14B···O14^v^	0.88	2.17	2.947 (5)	147.
N15—H15A···O25^v^	0.88	2.17	3.034 (5)	169.
N15—H15B···O22^viii^	0.88	2.05	2.911 (5)	167.

Symmetry codes: (i) −*x*+1, −*y*+1, −*z*+1; (ii) −*x*+1/2, *y*−1/2, −*z*+1/2; (iii) *x*−1, *y*, *z*; (iv) −*x*+1/2, *y*+1/2, −*z*+1/2; (v) −*x*+1, −*y*+1, −*z*; (vi) *x*−1/2, −*y*+3/2, *z*+1/2; (vii) −*x*+3/2, *y*+1/2, −*z*+1/2; (viii) −*x*+3/2, *y*−1/2, −*z*+1/2.
